# Diffuse Myocardial Metastases From Neuroendocrine Malignancy

**DOI:** 10.7759/cureus.106646

**Published:** 2026-04-08

**Authors:** Ryan Johnson, Nikhil Kanthala, Christine Burgert-Lon, Mandip Gakhal

**Affiliations:** 1 Diagnostic Radiology, Christiana Care Health System, Newark, USA; 2 Neuroradiology, Mid Atlantic Radiology Consultants, Media, USA; 3 Pathology, Christiana Care Health System, Newark, USA

**Keywords:** cardiac metastases, cardiac mri, myocardial thickening, neuroendocrine tumor, thymus

## Abstract

Neuroendocrine tumors of the thymus (NETT) are rare anterior mediastinal tumors known for their aggressive metastatic potential. This case report describes a middle-aged patient who presented with dyspnea. Multimodality imaging, including computed tomography (CT), transthoracic echocardiography (TTE), cardiac magnetic resonance imaging (MRI), and Gallium-68 DOTATATE positron emission tomography (PET), was used to suggest a diagnosis of NETT, which was confirmed by biopsies of the mediastinal mass and myocardium. Chemotherapy and octreotide were initiated, and follow-up DOTATATE PET demonstrated a partial treatment response. In the absence of appropriate clinical history and ancillary imaging findings, such as an adjacent mediastinal mass, the cardiac imaging features observed in this case (biventricular enlargement with patchy, diffuse myocardial hyperenhancement) may present a diagnostic dilemma because they are nonspecific. This case highlights the importance of distinguishing myocardial metastasis from hypertrophic cardiomyopathy (HCM) and infiltrative cardiomyopathy.

## Introduction

Neuroendocrine tumors of the thymus (NETTs) are rare anterior mediastinal tumors [1] that were first described by Rosai and Higa in 1972 [2]. In 2017, the incidence of NETTs in the United States was reported to be 0.18 cases per 1,000,000 persons, with a predilection for males in the fourth to fifth decade of life [2]. Compared with primary neuroendocrine tumors (NETs) arising in other locations, NETTs tend to exhibit more aggressive behavior. They are reported to be malignant in over 80% of cases, in contrast to approximately 25% of lung carcinoids. They are associated with a poor prognosis due to frequent local recurrence and a high propensity for metastasis [2].

Metastatic involvement of the heart is 20-40 times more common than primary cardiac neoplasms, and autopsy studies of patients with known extracardiac malignancy report cardiac involvement in 10%-12% of cases [3]. Most cases of cardiac metastasis remain clinically silent and are often undetected unless identified on advanced imaging, such as magnetic resonance imaging (MRI) or positron emission tomography (PET), or when symptoms related to heart failure develop. In malignancies arising in adjacent structures, including lung, breast, or esophageal carcinoma, direct extension is the most common mechanism of cardiac involvement [4]. Other pathways of spread include lymphatic, venous, or arterial dissemination [3].

Cardiac metastatic disease does not have a characteristic imaging appearance on MRI. Metastases typically demonstrate low signal intensity on T1-weighted images, high signal intensity on T2-weighted images, and variable enhancement patterns following intravenous contrast administration [3]. Given the lack of specificity, the differential diagnosis could include other entities such as hypertrophic cardiomyopathy (HCM), hypertensive cardiomyopathy, amyloidosis, and other infiltrative cardiomyopathies. Therefore, careful integration of clinical presentation, laboratory findings, and additional imaging studies is essential during interpretation.

A review of the literature reveals only two reported cases of NETs demonstrating diffuse cardiac metastases: one symptomatic case involving a lung carcinoid and one asymptomatic case secondary to a NETT [1,5]. The present case is unique in that it describes diffuse cardiac metastases from a NETT in a patient presenting with clinical features of heart failure.

## Case presentation

A 52-year-old man with a history of hypertension sought emergency care for two months of abdominal pain, dyspnea, and orthopnea. His vital signs showed a respiratory rate of 25 breaths per minute and 96% oxygen saturation; other vitals were normal. Physical examination revealed mild conversational dyspnea, with unremarkable lung and heart auscultation. An electrocardiogram indicated nonspecific atrial enlargement and T-wave abnormalities.

**Figure 1:**
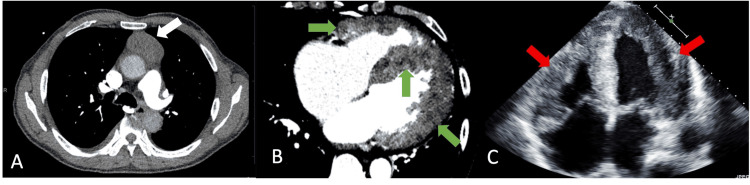
Admission CT scans and TTE (A) Axial pulmonary artery CTA demonstrates an anterior mediastinal mass of soft tissue attenuation (white arrow) measuring 5.2 x 2.8 x 5.2 cm (AP x TV x CC) at the expected location of the thymus (smaller mass not pictured). (B) Axial CT of the abdomen and pelvis with contrast performed on the same day, zoomed in on the heart, demonstrates diffuse myocardial thickening of the right and left ventricles with heterogeneous enhancement (green arrows). (C) Apical four-chamber view TTE demonstrates moderate to severe right ventricular hypertrophy and severe left ventricular hypertrophy (red arrows). Left ventricular ejection fraction was approximately 20%. Left ventricular myocardium was reported to have a speckled appearance, suggesting a diffuse infiltrative process. CT: computed tomography, TTE: transthoracic echocardiography, CTA: computed tomography angiography, AP: anteroposterior, TV: transverse, CC: craniocaudal.

Given concern for pulmonary embolus, a computed tomographic angiography (CTA) of the chest was performed. It revealed two anterior mediastinal masses (5.2 x 2.8 x 5.2 cm and 2.1 x 1.2 x 2.1 cm) in the anterior mediastinum, without local invasion, and diffuse thickening with heterogeneous attenuation of the myocardium (Figures 1A, 1B). Transthoracic echocardiogram (TTE) (Figure 1C) demonstrated moderate to severe biventricular hypertrophy, myocardial speckling, pulmonary artery pressure of 55 mmHg, severe left atrial enlargement, mild mitral valve regurgitation, and a resting global left ventricular ejection fraction of 20%. There was severe global left ventricular hypokinesia with apical sparing and a small pericardial effusion. Laboratory testing revealed an elevated chromogranin A of 107.1 ng/mL (0.0-101.8 ng/mL) and a normal serotonin of 96 ng/mL (31-207 ng/mL).

**Figure 2:**
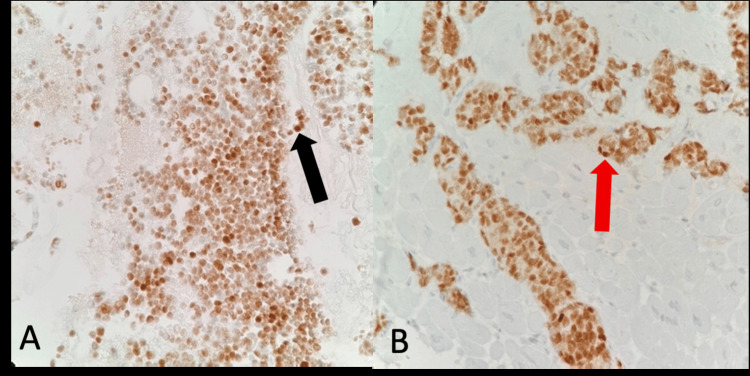
Pathology results from CT-guided mediastinal biopsy and cardiac catheterization myocardial biopsy (A) Insulinoma-associated protein 1 (INSM1) immunohistochemical stain of the cell block material from the mediastinal mass biopsy, demonstrating strong nuclear staining in the tumor cells (400X), indicative of a neuroendocrine tumor (black arrow). (B) INSM1 immunohistochemical stain of cardiac muscle showing strong nuclear staining in the tumor cells (400X), consistent with neuroendocrine metastases (red arrow). CT: computed tomography.

A CT-guided biopsy of the larger anterior mediastinal mass confirmed a well-differentiated NET favoring atypical carcinoid (Figure 2A). Cardiac catheterization with myocardial biopsy confirmed neuroendocrine metastases without evidence of myocardial fibrosis or other infiltrative processes (Figure 2B).

**Figure 3:**
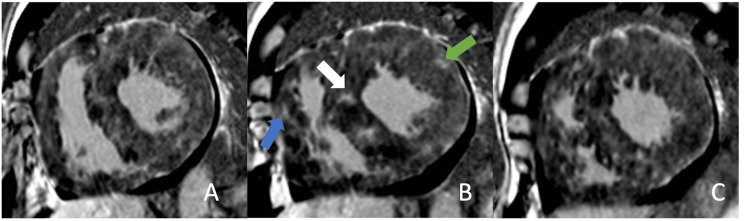
Cardiac MRI Short-axis delayed post-contrast phase-sensitive inversion recovery (PSIR) at the (A) basal, (B) mid-cavity, and (C) apical levels. Extensive biventricular myocardial thickening from the base to apex with foci of patchy, nodular, and hazy abnormal delayed myocardial hyperenhancement is present. These findings are demonstrated in the mid cavity right ventricle (blue arrow), septum (white arrow), and left ventricle (green arrow). Similar findings are also visualized in the basal and apical segments.

A regadenoson stress single-photon emission computed tomography (SPECT) study showed severe global left ventricular hypokinesia but no perfusion defect. Cardiac MRI revealed extensive diffuse biventricular myocardial thickening with multifocal patchy, nodular, and hazy abnormal delayed myocardial hyperenhancement throughout both ventricles (Figure 3). Immediate post-contrast T1-weighted images were not obtained, as the study followed a cardiomyopathy protocol, not a cardiac mass workup.

**Figure 4:**
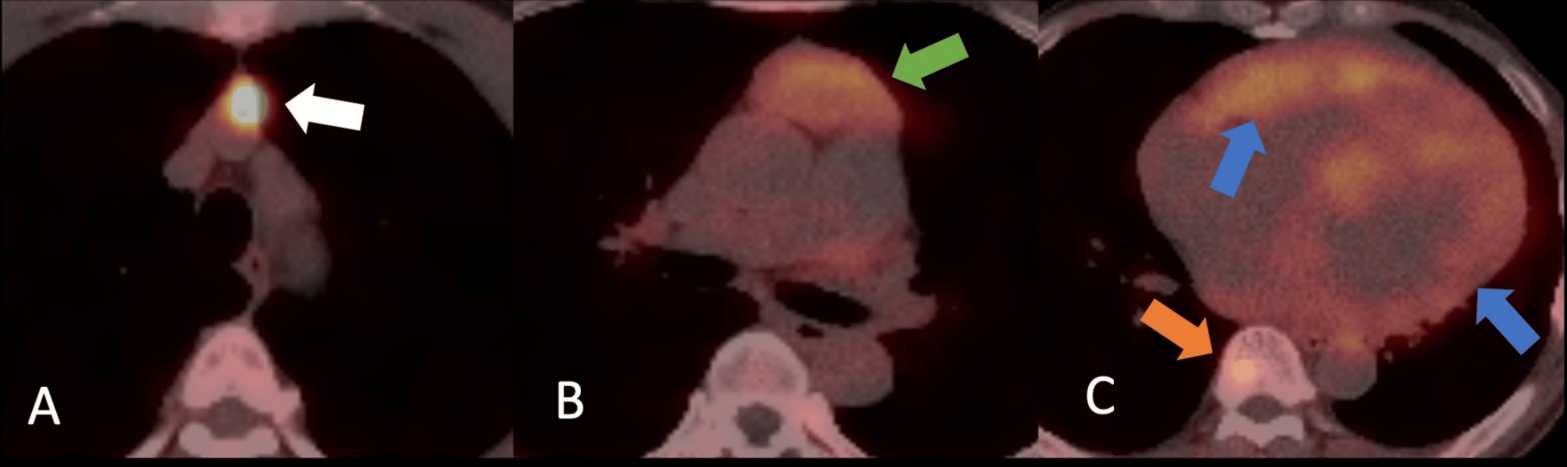
Initial staging DOTATATE PET/CT Images from the pretreatment gallium-68 DOTATATE PET/CT were analyzed, with standardized uptake values (SUV) reported as the maximum values normalized to lean body mass; the mean liver SUV was 4.9. (A) Axial image at the level of the thymus in the anterior mediastinum showing a mass (white arrow) with increased metabolic activity (SUV 17). (B) Slightly inferior, also in the anterior mediastinum, showing an additional mass (green arrow) with increased metabolic activity (SUV 4.3). These anterior mediastinal masses are consistent with neuroendocrine neoplasm. (C) Diffusely increased uptake in the left and right ventricles (blue arrows) (SUV 4.9). A focus of increased metabolic activity in the vertebral body of T9 (orange arrow) (SUV 2.3). PET/CT: positron emission tomography/computed tomography.

Gallium-68 DOTATATE PET/CT (standard uptake value (SUV) max reported utilizing lean body mass, mean SUV liver value 4.9) identified increased uptake in the two anterior mediastinal thymic lesions. The smaller lesion had an SUV max of 17, and the larger biopsied lesion had an SUV max of 4.3. One lesion was presumed primary, the other a local metastatic lymph node or a secondary lesion. No abnormal uptake was noted in the pancreas or gastrointestinal tract, ruling out other primary NET sites. Diffuse patchy increased myocardial uptake (SUV max 4.9) was consistent with diffuse myocardial metastases. Multifocal sclerotic lesions in the cervical, thoracic, and lumbar spine, bilateral ribs, scapula, and iliac wings (SUV max 3.5) were consistent with osseous metastases (Figures 4A-4C).

Chemotherapy with capecitabine (Xeloda) and temozolomide (Temodar), and octreotide for prophylactic carcinoid toxicity, was initiated. At 21 months of follow-up, DOTATATE PET/CT showed imaging signs of partial metabolic treatment response.

Two years post-presentation, the patient returned to the emergency department with worsening shortness of breath and severe hypotension. Emergency TTE and CTA revealed a large pericardial effusion, prompting pericardiocentesis due to suspected tamponade physiology, with 1 L of straw-colored fluid removed. The patient was discharged to hospice.

## Discussion

This case highlights multimodality imaging findings of diffuse cardiac metastases from a rare mediastinal neoplasm, initially presenting as heart failure and resembling infiltrative and hypertrophic cardiomyopathies.

Neuroendocrine tumors have a 2%-4% incidence of cardiac metastasis, typically indicating advanced disease [6]. Cardiac metastases often present with nonspecific symptoms like valvular dysfunction, arrhythmias, pericardial effusions, or intracardiac blood flow obstruction, or they can be asymptomatic, leading to incidental discovery at an advanced stage, limiting treatment options and worsening prognosis [7].

NETTs are rare mediastinal tumors, accounting for about 0.4% of all NETs and less than 5% of anterior mediastinal malignancies [8]. NETTs are generally aggressive, with over 80% being malignant [2]. They are classified as typical carcinoid, atypical carcinoid, large cell neuroendocrine carcinoma, and small cell neuroendocrine carcinoma [8]. Metastases commonly affect the liver, brain, and bone; cardiac involvement is rare. Kunz et al. reported higher Ki67 index levels in NETs with myocardial metastasis, indicating more aggressive behavior [9], which is consistent with our patient's biopsy results (Ki67 up to 20%). Cardiac metastasis usually occurs in advanced disease with other systemic metastases. Notably, our case showed no findings consistent with carcinoid heart disease, which is typically caused by high serotonin levels leading to cardiac fibrosis [10]. Foregut carcinoid tumors often lack the enzyme (aromatic amino-acid decarboxylase) needed to convert tryptophan to serotonin, which may explain the absence of fibrosis on cardiac biopsy in this case, contributing to the delayed myocardial enhancement without the full clinical picture of carcinoid heart disease [11].

Tumors can metastasize to the heart and pericardium via four routes: lymphatic, hematogenous, direct extension, and intracavitary diffusion [12]. Myocardial metastases affect the right ventricle in 40%, the left ventricle in 53%, and the interventricular septum in 7% [9]. Direct extension to the heart is more common with lung, breast, and esophageal cancers [4]. While a 2020 report described diffuse myocardial involvement by NETT and a 2021 report noted direct myocardial extension from mediastinal carcinoid [13,14], direct extension was not observed in our case, despite the NETT's proximity to the heart, most suggestive of hematogenous spread.

Diagnosis relies on combined imaging techniques and histological analysis from endomyocardial biopsy or post-mortem evaluation [15,4]. In our case, echocardiography showed a small pericardial effusion, hypertrophy, and global hypokinesis of the left ventricle. In cases with clinical "red flags" (e.g., malignancy history) or imaging "red flags" (e.g., concomitant right ventricular hypertrophy, atypical left ventricular hypertrophy, or pericardial effusion), cardiac MRI can refine the differential diagnosis [16].

The differential diagnosis included HCM, cardiac amyloidosis, cardiac lymphoma, and diffuse myocardial metastases. HCM can present with diffuse biventricular myocardial thickening and patchy, nodular, or hazy abnormal delayed myocardial enhancement. However, the presence of pericardial effusion, reduced ejection fraction, and a known adjacent neuroendocrine tumor made this diagnosis less likely [17,18]. Cardiac amyloidosis also presents with myocardial thickening and variable delayed myocardial hyperenhancement. However, ancillary findings like diffuse lack of myocardial signal nulling, relatively hypointense blood pool on delayed post-contrast imaging, and atrial wall thickening/enhancement, typically seen in amyloidosis, were absent [18]. T1-mapping and extracellular volume (ECV) values, often significantly elevated in cardiac amyloidosis, were not available for this case. Cardiac lymphoma presents with diverse imaging manifestations, including those seen here, but a second malignancy is less probable given a known primary [3]. A definitive diagnosis was achieved via myocardial biopsy.

## Conclusions

We report a case of biopsy-proven diffuse cardiac metastases from a neuroendocrine tumor, presumably of thymic origin, presenting with heart failure symptoms and mimicking diffuse myocardial hypertrophy and infiltrative cardiomyopathy. This case underscores the importance of considering neoplastic etiologies in the imaging differential diagnosis for diffuse myocardial thickening or lesions when imaging features suggest an infiltrative or mass-like process.
